# An encounter with the mild side of *LARS2*-associated Perrault syndrome and its implications on the diagnostic odyssey

**DOI:** 10.1038/s41431-023-01285-0

**Published:** 2023-01-24

**Authors:** Barbara Vona

**Affiliations:** 1 Institute of Human Genetics, University Medical Center Göttingen, Göttingen, Germany; 2 Institute for Auditory Neuroscience and InnerEarLab, University Medical Center Göttingen, Göttingen, Germany

**Keywords:** Genetic testing, Infertility, Molecular biology

The diagnostic odyssey is an agonizingly long journey often experienced by affected individuals and their loved ones on the quest toward answers for their rare disease. Since entering the post-genomic era, the diagnostic odyssey and what it entails has sharply come into focus, as can be seen from a quick query of the phrase “diagnostic odyssey” in PubMed. As of 31st December 2022, there are over 300 hits, with most of them from the last 5–6 years as molecular genetic testing becomes increasingly mainstream. Finding ways to shortcut the time to diagnosis is crucial and is the tireless mission of many colleagues in the field.

Among the many contributing factors for delayed diagnoses include deviations in the order and appearance of clinical features, as well as the patient experience in navigating multiple specialties and other barriers to diagnosis [1]. As the field uncovers new and expands upon previously described hereditary syndromes, it is tasked with delineating the clinical order in which phenotypes appear, their evolution over time, and recognition of extremes in the phenotypic spectrum. In this issue of the *European Journal of Human Genetics*, Neyroud et al. [2] describe an atypical presentation of Perrault syndrome due to biallelic variants in *LARS2*, initially appearing as isolated premature ovarian insufficiency, serving as an excellent example of the power of a genetic diagnosis in light of an initially divergent presentation.

Perrault syndrome is a rare autosomal recessive disorder classified according to two clinical sub-groups [3]. Types 1 and 2 include sensorineural hearing loss in both males and females, usually starting at birth or early childhood and premature ovarian insufficiency in females with a 46, XX karyotype. Type 2 appears as a variable and complex neurological syndrome. Hearing loss is often described as progressive and in most extreme cases, can begin as mild and advance to severe or profound throughout life. A clinical diagnosis of Perrault syndrome is impossible to achieve in certain contexts, for example, in sporadic affected males and prepubescent females before the onset of premature ovarian insufficiency, whereby, in the absence of neurological symptoms, their features are clinically indistinguishable from non-syndromic hearing loss. Undiagnosed prepubescent individuals with Perrault syndrome who pursue genetic testing when isolated hearing impairment is present may uncover a hidden or unrecognized syndrome, improving patient management, yielding predictive value of potential co-morbidities and may identify additional individuals in the family if segregation testing of the variant(s) is performed. If diagnosed early, the benefit of a prediction of future ovarian dysfunction can be made, improving clinical outcomes and possibly allowing oocyte cryopreservation, permitting ovarian reserve.

*LARS2* (OMIM: *604544) was identified as a Perrault syndrome-associated gene in 2013 (PRLTS4, OMIM: #615300) [4]. In aggregate, nearly a dozen genes are currently linked to Perrault syndrome. *LARS2* is a nuclear gene encoding a mitochondrial leucyl-tRNA synthetase that charges mitochondrial tRNAs with leucine. Mitochondrial translation is a highly energy intensive process. Dysfunction of mitochondrial enzymes can impair protein synthesis, cause energy deficits, increase reactive oxygen species with a tissue-specific effect and accelerate or alter timing of apoptosis [4]. Furthermore, the effects of other genetic and environmental factors, including modifiers, cannot be excluded as contributors to the high degree of clinical heterogeneity, even among family members segregating the same pathogenic variants, similar to other amino-acyl tRNA synthetase disorders.

Exome sequencing was performed in two French sisters whose sole complaint was premature ovarian insufficiency. Unexpectedly, compound heterozygous variants, both residing in the highly conserved catalytic domain of *LARS2*, were uncovered, including a previously described pathogenic variant (NM_015340.3:c.1565 C > A, NP_056155.1:p.(Thr522Asn)) and a novel variant (NM_015340.3:c.1670A > G, NP_056155.1:p.(Tyr557Cys)) that was classified as pathogenic following functional studies. The novel variant significantly affected leucylation efficiency to such an extent that the degree of *LARS2* dysfunction did not correlate with the relatively mild phenotype. What was particularly unexpected was the lack of overt hearing impairment in either individual. The age at genetic diagnosis was 21 years for the proband and 16 years for her sister. While the age at genetic diagnosis is not particularly exceptional, as other reports in the literature describe individuals diagnosed at even older ages, one has to wonder if the initially reported subjectively normal hearing caused significant delays in achieving a genetic diagnosis.

Following molecular genetic diagnosis, both individuals recalled some minor difficulties in understanding conversations, retrospectively admitting that they have to ask people to repeat themselves but they did not think anything particularly unusual. Audiological assessment showed mild, bilateral, low-frequency hearing loss in both sisters. Hereditary low-frequency hearing loss is rather rare, with only a few genes associated with an upward sloping audiogram profile [5]. Interestingly, a school nurse recommended further hearing assessment of the younger sister following the identification of mild hearing loss at the age of 9 years that was not pursued but could have possibly triggered genetic diagnostic testing 7 years earlier. This may have potentially prompted segregation testing of the *LARS2* variants and diagnosed Perrault syndrome years before the onset of premature ovarian insufficiency in the proband and would have been helpful for improving overall management of progressive co-morbidities in both individuals. Even mild hearing loss can pose significant challenges to individuals [6] and should justify initiating genetic testing when environmental factors can be excluded. Going forward, audiological follow-up will be particularly important to monitor progressive hearing loss.

Neyroud et al. described the first report of a patient with Perrault syndrome to seek clinical consultation due to premature ovarian insufficiency instead of hearing loss [2]. It underscores new considerations for genetic counselling of families with *LARS2*-related Perrault syndrome with recognition of the presenting symptom as isolated premature ovarian insufficiency, broadening the phenotypic spectrum and highlighting the strong potential for genetic testing to provide insight into other co-morbidities, like hearing loss, before progression. Nearly 10 years have passed since the initial gene discovery linking *LARS2* to Perrault syndrome [4]. Considering its rarity and extreme clinical heterogeneity, it is clear that much more work remains to limit the diagnostic odyssey experienced by these families and to recognize clinical extremes at the mild end of the spectrum.

## References

[1] Evans WR (2018). Dare to think rare: diagnostic delay and rare diseases. Br J Gen Pr.

[2] Neyroud AS, Rudinger-Thirion J, Frugier M, Riley LG, Bidet M, Akloul L, et al. LARS2 variants can present as premature ovarian insufficiency in the absence of overt hearing loss. Eur J Hum Genet. 2022. 10.1038/s41431-022-01252-1 10.1038/s41431-022-01252-1 PMC10133321 36450801

[3] Newman WG, Friedman TB, Conway GS, Demain LA. Perrault Syndrome. In: Adam MP, Everman DB, Mirzaa GM, Pagon RA, Wallace SE, Bean LJ, et al., editors. GeneReviews® [Internet]. Seattle (WA): University of Washington, Seattle; 1993 [cited 2022 Dec]. http://www.ncbi.nlm.nih.gov/books/NBK242617/25254289

[4] Pierce SB, Gersak K, Michaelson-Cohen R, Walsh T, Lee MK, Malach D (2013). Mutations in LARS2, encoding mitochondrial leucyl-tRNA synthetase, lead to premature ovarian failure and hearing loss in Perrault syndrome. Am J Hum Genet.

[5] Demain LAM, Urquhart JE, O’Sullivan J, Williams SG, Bhaskar SS, Jenkinson EM (2017). Expanding the genotypic spectrum of Perrault syndrome. Clin Genet.

[6] GBD 2019 Hearing Loss Collaborators. (2021). Hearing loss prevalence and years lived with disability, 1990–2019: findings from the Global Burden of Disease Study 2019. Lancet.

